# Special Issue “Wet Chemical Synthesis of Functional Nanomaterials”

**DOI:** 10.3390/nano11041044

**Published:** 2021-04-19

**Authors:** Enrico Della Gaspera

**Affiliations:** School of Science, RMIT University, Melbourne, VIC 3000, Australia; enrico.dellagaspera@rmit.edu.au

“Wet chemical synthesis, also called solution processing, represents an accessible, versatile, and powerful approach for synthesizing materials with excellent control of their structural, chemical, and physical properties”. This was the opening sentence for the Special Issue titled: Wet Chemical Synthesis of Functional Nanomaterials. This simple, short statement describes a vast, diverse, and rapidly developing field: it encompasses different types of materials, different types of synthesis methods, and also different types of applications. Such a broad spectrum of features highlights the importance and impact that wet chemical synthesis has for the nanoscience and nanotechnology communities. In fact, it provides a means to synthesize nanomaterials with precise control of their morphological, structural, and chemical properties (such as size, shape, composition, crystallinity, and surface chemistry), which consequently affect their functional properties (optical, electrical, catalytic, magnetic, etc.). However, wet chemical synthesis has traditionally been regarded as a “dirty” process, because it usually does not provide the same quality and purity if compared with vacuum based deposition methods, such as sputtering and thermal or electron-beam evaporation. This (mis)conception regarding solution processing is slowly being corrected, because immense progress has been reported on the synthesis and application of nanomaterials using wet chemistry methods. Many reports have been released demonstrating performances of solution-processed materials that are on par, or even better than those achieved with vacuum based depositions. This Special Issue aimed to provide a forum to present some of the latest results in the area of synthesis, characterization and application of functional nanomaterials which are synthesized using wet chemical methods. With five articles and one review covering polymer oxide composites for magnetic applications [1], TiO_2_/Au nanostructured films for hydrogen sensors [2], mixed oxide nanoparticles derived from sacrificial metal organic frameworks [3], highly doped metal oxide colloidal nanocrystals for plasmonics [4], sol-gel derived mixed alkali niobate coatings for piezoelectric micro-devices [5], and solution-processed thin films transistors based on oxide heterojunctions [6], this special issue is a perfect representation of the breadth and impact of this dynamic, multidisciplinary, and impactful research field.

Magnetic composites based on widely available materials (without the use of rare earth elements) are highly sought after for countless applications including hard drives, magnetic brakes, motors, and MRI scanners. The ability to use earth-abundant elements coupled with simple and straightforward fabrication methods will constitute a major progress in this field. Sirisathitkul and co-workers developed magnetic composites based on barium hexaferrite magnetic particles dispersed within a polymeric matrix, and produced a composite material with the potential to be fabricated via 3D printing, owing to the mechanical properties achieved by the combination of inorganic fillers and an organic matrix [1]. High coercivity values of over 2000 Oe were demonstrated, proving the high performance of such composites, leading the way for further studies into the additive manufacturing of such composites.

Gas sensors based on optical detection rather than electrical have emerged in the past couple of decades due to their improved performance, especially in their ability to distinguish interfering gases through wavelength-dependent response. This is particularly true for plasmonic sensors, where the optical signal is attributed to changes in the plasmonic resonance of metal nanoparticles or gratings. Gazzola et al. presented a comparison between two types of plasmonic sensors for hydrogen detection based on solution-processed TiO_2_ layers: a grating-coupled surface plasmon resonance (SPR) sensor and a localized SPR sensor based on Au nanoparticles [2]. Both sensors responded to hydrogen gas, and the mechanism was the dissociation of an H_2_ molecule on the TiO_2_ surface, promoting electron injection into the semiconducting layer. However, the presence of small Au nanoparticles has also been proven to enhance the sensing performance due to a photocatalytic effect, that is absent in the grating-based sensors, elucidating the sensing mechanism for these plasmonic sensors and laying the foundation for further studies in this field.

The synthesis of nanomaterials with novel or improved properties is the basis of advancement in technological applications. In this view, Soldatov and coworkers presented the synthesis of zinc–cobalt mixed oxide nanoparticles using ZIF-8 (zeolitic imidazolate frameworks) metal organic frameworks (MOFs) as sacrificial agents [3]. The authors observed doping of cobalt into zinc oxide, as well as the formation of spinel Co_3_O_4_ depending on composition, and they also discovered that the addition of a silicon source could provide a protective SiO_2_ layer on the surface of the nanoparticles. Importantly, wurtzite zinc oxide could be synthesized with oxygen vacancies, opening applications in catalysis and sensing. This work therefore showed a convenient approach to synthesize a family of nanomaterials with tunable properties.

Nanoparticles suspended in solutions as stable colloids have been intensively studied as they can constitute building blocks for printed electronic devices including circuitry, sensors, solar cells, LEDs, and electrochromics. Kendall et al. presented a colloidal synthesis for tin dioxide nanocrystals doped with fluorine [4]. F-doped SnO_2_ (FTO) is one of the main transparent conducting oxides used in optoelectronics and smart windows, however, its processing conditions are limited to thin film coatings deposited at high temperatures. This work describes the synthesis of small (<5 nm) FTO nanoparticles showing composition-dependent optical properties including blue luminescence and a surface plasmon resonance due to the presence of free carriers, expanding the range of available plasmonic metal oxide nanocrystals.

Piezoelectric materials have many applications in sensors, actuators, motors, and high-power sources, just to name a few. The industry standard for piezoelectric materials is lead zirconate titanate, but there is an urgent need for alternatives due to the environmental issues of using lead. Vilarinho and co-workers presented a study on K_0.5_Na_0.5_NbO_3_ thin films synthesized via the sol-gel method [5]. They investigated the role of the precursor concentration and stoichiometry on the crystallinity, grain size, and orientation, and related these structural and morphological properties to their functional piezoelectric performance, unraveling the complex interplay between all of these indicators. This study is therefore a step forward in the replacement of lead-based piezoelectric micro-devices.

Thin-film transistors are ubiquitously used in consumer electronics, especially flat panel displays. Metal oxide-based transistors are more appealing than conventional silicon devices because of their high mobility, high transparency, and excellent performance. In their review article, Li et al. presented a detailed overview of the recent advances in solution-processed thin-film transistors based on oxide heterojunctions [6]. The authors specifically focus on two key approaches to enhance the performance of these transistors: electrical-property modulation and improved mobility via formation of a 2D electron gas, providing an understanding and stimuli towards further research into high-performance oxide transistors.

I hope that the readers of Nanomaterials find this Special Issue appealing and informative, and I also hope that they might gain insights and inspirations for their future research efforts.
